# Prenatal exposure to acrylamide and neuropsychiatric disorders: a biomarker-based birth cohort study with 34 years of follow-up

**DOI:** 10.1016/j.eclinm.2026.104286

**Published:** 2026-10-26

**Authors:** Damian Chandia-Poblete, Stéphane Tuffier, Efstathios Vryonidis, Thorhallur Ingi Halldorsson, Anne Ahrendt Bjerregaard, Marin Strøm, Dorte Rytter, Bodil Hammer Bech, Tine Brink Henriksen, Sjurdur Frodi Olsen, Margareta Törnqvist, Marie Pedersen

**Affiliations:** a Department of Public Health, University of Copenhagen, Copenhagen, Denmark; b Department of Environmental Science, Stockholm University, Stockholm, Sweden; c Department for Congenital Disorders, Statens Serum Institute, Copenhagen, Denmark; d Faculty of Food Science and Nutrition, University of Iceland, Reykjavik, Iceland; e Centre for Clinical Research and Prevention, Copenhagen University Hospital - Bispebjerg and Frederiksberg Hospital, Copenhagen, Denmark; f Faculty of Health Sciences, University of the Faroe Islands, Torshavn, Denmark; g Department of Public Health, Aarhus University, Denmark; h Department of Clinical Medicine, Paediatrics, Health, Aarhus University, Denmark; i Department of Nutrition, Harvard School of Public Health, Boston, United States

**Keywords:** Anxiety, Depression, Developmental origins of health and disease, Dietary habits, Haemoglobin adducts

## Abstract

**Background:**

The widespread exposure to acrylamide from diet and tobacco smoke during prenatal development is of concern as acrylamide is neurotoxic and potentially contributes to the development of neuropsychiatric disorders. We aimed to examine associations between prenatal acrylamide exposure and neuropsychiatric outcomes.

**Methods:**

In this biomarker-based study of offspring from the Danish Foetal Origins 1988–1989 birth cohort, pregnant women were enrolled at gestational week 30 at a routine antenatal clinic at Aarhus Hospital, Denmark. Liveborn singletons with available exposure and outcome data were included. The current study was conducted from 2022 to 2026. Prenatal acrylamide exposure was quantified using haemoglobin adducts (HbAA) in maternal blood collected during pregnancy. Incident cases of depression, anxiety, attention-deficit/hyperactivity disorder (ADHD), co-occurring ADHD and autism spectrum disorder (ASD), as a composite outcome, and any neuropsychiatric disorders were identified from national diagnostic and prescription registries in the first 34 years of life (1988–2023). The associations were estimated using adjusted log-linear Poisson regression models.

**Findings:**

Among 861 offspring, 146 cases of depression, 72 of anxiety, 45 of ADHD, 53 of co-occurring ADHD and ASD, and 150 cases of any neuropsychiatric diagnosis occurred during follow-up. Median (P5–P95) HbAA was 93 (45–250) pmol/g Hb and maternal active smoking during pregnancy was common (42%). Each 10-pmol/g Hb increase in HbAA was associated with IRRs of 1.02 (95% CI 0.99–1.05) for depression and 1.03 (0.99–1.07) for anxiety. High vs. low exposure (≥P75 = 146 pmol/g Hb, vs. <P75) was associated with increased risks of depression (IRR 1.64, 1.05–2.56) and anxiety (IRR 2.29, 1.23–4.25). The estimates for ADHD (1.56, 0.73–3.32), co-occurring ADHD and ASD (1.49, 0.73–3.02) and any neuropsychiatric diagnosis (1.41, 0.90–2.22) showed non-significant increased risk.

**Interpretation:**

The findings support an association between prenatal acrylamide exposure and increased incidence of depression and anxiety in adolescence and adulthood. These findings require replication in cohorts with repeated exposure measurements of a range of dietary exposures to establish to what extent the prenatal exposure to acrylamide was responsible for the observed associations.

**Funding:**

10.13039/501100000781European Research Council, 10.13039/100012774Innovation Fund Denmark, 10.13039/100007398Danish Council for Strategic Research and 10.13039/501100009244Stockholm University.

**Research in context**

**Evidence before this study**

Acrylamide exposure from diet is widespread as acrylamide forms in a wide range of commonly consumed carbohydrate-containing foods and drinks during high temperature cooking. Tobacco smoke is another source of acrylamide exposure. The widespread dietary exposure to acrylamide is a significant public health concern, especially to pregnant women and their developing child, as acrylamide possess electrophilic reactivity, is neurotoxic, genotoxic, probably carcinogenic to humans, crosses the human placenta, restrict fetal growth, and may contribute to the development of neuropsychiatric disorders.

Experimental and occupational studies have provided a large body of evidence of acrylamide- induced neurotoxicity, including neurodevelopmental neurotoxicity and changes in brain structure, which has been complemented by emerging evidence from studies of dietary exposures to acrylamide and studies based on acrylamide quantified as haemoglobin adducts (HbAA) in children and adults. However, longitudinal prospective birth cohort studies of associations between prenatal exposure to acrylamide and neuropsychiatric disorders that occur later in life are lacking.

**Added value of this study**

To address this knowledge, we quantified prenatal exposure to acrylamide through measurement of HbAA in blood collected from pregnant women and examined the associations between prenatal exposure to acrylamide and incidence of neuropsychiatric disorders from birth to 34 years of age. The findings of our study indicated that higher prenatal exposure to acrylamide (both from diet separately and from smoking) was associated with increased incidence rate ratios for depression and anxiety, with a possibly higher risk at the upper range of exposure. The current study strengthens the evidence suggesting that prenatal exposure to acrylamide in the general population is associated with the development of depression and anxiety. The longitudinal study design with 34 years of follow-up of the offspring health following assessment of prenatal exposure establishes the temporal sequence between prenatal exposure to acrylamide and neuropsychiatric disorders, diminishing the possibility of reverse causality. Additional strengths include the ability to quantify Hb adducts from acrylamide in maternal blood collected during a critical window of development as part of a well-characterised birth cohort; to adjust for a wider range of confounders; and to link with high-quality registries allowing up to 34 years of follow-up of the full population for detailed health assessment. The HbAA levels reflect internal dose over the past four months accumulating from all sources of exposures and provide a more accurate measure than estimates based on questionnaires as the concentrations of acrylamide in food depends on many factors such as food preparation which are difficult to capture accurately in questionnaires. A limitation was that the HbAA levels were measured only once and measuring exposure over a single period during prenatal development and having a long follow-up period, may not fully capture later changes in maternal and offspring acrylamide-related dietary or lifestyle habits. We also cannot rule out residual confounding by behavioural factors associated with high acrylamide exposure, or by maternal mental health, despite extensive adjustments and modelling. Our findings indicated that HbAA may be an independent risk factor for depression and anxiety when controlling for maternal smoking and other confounders, suggesting that nonsmokers and the offspring of mothers with acrylamide-rich diet face increased risk of developing depression and anxiety.

**Implications of all the available evidence**

The findings from the present study extend the existing evidence from experimental, occupational and epidemiological studies of acrylamide-induced neurotoxicity, and supports an association between prenatal exposure to acrylamide and certain subsequent onset of neuropsychiatric outcomes. Overall, the evidence supports the hypothesis that prenatal exposure to acrylamide in the general population contribute to increased risk of neuropsychiatric disorders. These findings may have profound implications for public health given the large burden associated with these disorders, and the ubiquity of acrylamide exposure in our society.

## Introduction

Acrylamide forms at high-temperature processing in multiple commonly consumed foods and drinks containing asparagine and reducing sugars at high-temperature processing.^1^, ^2^ The concentrations of acrylamide are high in potato chips, fried potatoes, biscuits, bread, coffee, and baked cereals, but the concentrations vary widely, even within the same food products, because the formation of acrylamide depends on multiple factors including cooking methods, food composition and storage.^3^, ^4^, ^5^, ^6^ Quantification of dietary intake of acrylamide based on food-frequency questionnaires (FFQs) or recalls are uncertain^7^ whereas determination of haemoglobin (Hb) adducts from acrylamide (HbAA) (i.e., stable chemical product formed when acrylamide covalently bind to the amino acid residues of Hb inside red blood cells) provides an accurate measure of internal dose to acrylamide.^8^ The HbAA levels reflect exposure accumulated over the past four months from all exposures sources including dietary and tobacco smoke.^9^

The widespread dietary exposure to acrylamide is a significant public health concern because acrylamide possess electrophilic reactivity, is neuro-, repro- and genotoxic and, notably, acrylamide has been classified as Group 2A “probable human carcinogen” by the International Agency of Cancer Research.^10^ Acrylamide crosses the human placenta,^11^ has been detected in human second trimester amniotic fluid,^12^ and has been found to be associated with restricted fetal growth.^13^, ^14^ Higher HbAA levels have also been associated with smaller birth head circumference,^13^, ^15^ which has been linked with lower cognitive function and neurological development later in life.^16^, ^17^ Higher HbAA levels in maternal blood collected from pregnant women have been reported to be highly correlated with higher HbAA cord blood levels as well as with higher maternal intake of acrylamide-rich foods and maternal active smoking during pregnancy.^15^, ^18^

Exposure during the early life, starting during prenatal development, is increasingly recognised as a critical period for the origin and emergence of mental health vulnerabilities and multiple mechanisms have been proposed to play a role.^19^ The biological plausibility that prenatal exposure to acrylamide may interfere with critical stages of prenatal brain development,^20^, ^21^ which may lead to long-term consequences, is supported by a large body of evidence of neurotoxicity from mechanistic and occupational studies,^20^, ^21^, ^22^, ^23^, ^24^, ^25^, ^26^, ^27^ as well as sparse suggestive evidence from observational studies of exposure to acrylamide during later life stages of the general population.^24^, ^28^, ^29^, ^30^

Epidemiologic evidence suggests that even low-level acrylamide exposure may be associated with adverse cognitive, developmental, and affective outcomes, including educational support needs in children, depressive symptoms in adults, and cognitive decline in elderly men.^24^, ^28^, ^29^, ^30^ The available evidence comes almost exclusively from cross-sectional studies of adults or from parent-reported unspecific outcomes in children. With exposure and outcomes being measured at the same time, neither the temporal sequence nor the possibility of reverse causation can be resolved. Furthermore, exposure to acrylamide, estimated by FFQ, is uncertain given that the acrylamide concentration varies widely within the same food item depending on the food composition and how it is prepared. To our knowledge, no study has so far quantified exposure to acrylamide during prenatal development, although experimental evidence points to the developing brain as the most vulnerable target, and evaluated the associations with risk of neuropsychiatric disorders later in life. Because exposure to acrylamide is ubiquitous and modifiable, and because neuropsychiatric disorders carry a large burden on families and society, even a small risk at the population level, which if minimised, could translate into large public health benefits.

To address these knowledge gaps, we quantified prenatal exposure to acrylamide by measuring the HbAA levels in blood from pregnant women participating in a well-characterised birth cohort and examined associations between this biomarker of prenatal exposure to acrylamide, and incidence rates of neuropsychiatric disorders in the first 34 years of life of the offspring. We evaluated associations with clinically diagnosed depression, anxiety, attention-deficit/hyperactivity disorder (ADHD), co-occurrence of ADHD and autism spectrum disorder (ASD), as a composite outcome, and any neuropsychiatric diagnoses to cover the full range of neuropsychiatric disorders.

## Methods

### Study population

This study is based on the Danish Foetal Origins 1988–1989 (DaFO88) cohort.^31^ Briefly, women with a singleton pregnancy were recruited between 1988 and 1989. Eligible for recruitment were all women scheduled for a routine antenatal visit at a large antenatal clinic at Aarhus University Hospital, Denmark in gestational week 30. Out of 1212 eligible women, 991 (82%) enrolled in the DaFO88 cohort.^31^ Using the Danish civil registration number,^32^ cohort data were linked to nationwide registries to obtain sociodemographic, perinatal, diagnostic, mortality/emigration, and prescription data spanning 1988–2023 (prescriptions: 1995–2023). Detailed description of study population and linkage is found in Supplemental Material. Data on race or ethnicity were not collected, as this information was not collected in the cohort questionnaires in 1988–1989, and the population attending the clinic was almost exclusively of Danish origin. Follow-up through the Civil Registration System was complete apart from death and emigration, which were treated as censoring events, and diagnoses and redeemed prescriptions were obtained from registries with nationwide coverage.

### Ethics

The cohort study was approved by the Regional Scientific Ethical Committee of the county of Aarhus, Denmark and the Scientific Ethical Committee of the Capital Region of Denmark (reference no. 20070157 and H-19015014). Written consents were provided from the participating pregnant women prior to participation in the study. The measurement of acrylamide through quantification of Hb adducts in blood was approved by the Swedish Regional Ethical Authority (no. 2019-03181).

### Measurement of haemoglobin adducts biomarkers

We determined Hb adducts as biomarkers of internal dose of acrylamide and its reactive metabolite glycidamide, i.e., HbAA and HbGA, respectively. Further adduct levels from ethylene oxide (HbEO) were quantified to assess exposure to active and passive tobacco smoking during pregnancy in peripheral maternal blood. The blood samples used for adduct analysis were collected during the antenatal visit around gestational week 30 (n = 896), and at gestational week 37 (n = 3), then stored frozen until analysis.

The FI*R*E procedure was used for the measurement of haemoglobin adducts. The method is based on a modified Edman degradation and uses fluorescein isothiocyanate for the detachment of N-terminal Val adducts as fluorescein thiohydantoins (FTH). Thereafter, the FTHs are isolated with mixed mode anion exchange solid phase extraction and analysed by liquid chromatography–mass spectrometry.^18^, ^33^ In the current study, some modifications and optimizations were made to allow for the use of 96 deep well plates.^34^ Specific details for the Hb adduct analysis of this cohort is described elsewhere.^35^ The HbGA adduct was quantified in fewer samples than the HbAA adduct. This was due to difficulties with the current state of the analytical method with respect to the FTH analyte of HbGA, which appeared as a double peak, with lower overall signal-to-noise ratio than HbAA.

### Neuropsychiatric disorders definition

Offspring incident cases of depression, anxiety, and ADHD were identified from redemption of at least two prescriptions for medications indicated for these disorders or one diagnosis according to the International Classification of Diseases (ICD) codes, whichever occurred first from birth to the end of follow-up 31st of December 2023. Cases of ASD, or any neuropsychiatric diagnoses were identified from the first occurrence of a primary diagnosis, and we grouped all the neuropsychiatric diagnosis into one group to avoid small numbers as detailed in the Supplemental Material (Supplementary Table S1).

### Covariates

We identified potential confounding factors from literature and from biological knowledge and used a directed acyclic graph for an “a priori” selection of potential confounders (Supplementary Fig. S1).

We included and defined the following: maternal active smoking during pregnancy (yes, no), HbEO levels (pmol/g Hb), maternal alcohol intake during pregnancy (g/day) and maternal healthy eating index during pregnancy (continuous), maternal educational level at the time of birth (low: primary and lower secondary school, medium: general or vocational upper secondary education, high: university or other higher education), household income at the time of birth (quartiles as DKK/year), parity (0, 1+), sex and offspring gestational age (days).

### Statistical analysis

Descriptive analyses were performed in the case-complete dataset (N = 687). A descriptive comparison between excluded and included is presented in Supplementary Table S2. We report characteristics for all and after stratification by high and low prenatal exposure to acrylamide and maternal smoking status during pregnancy. We use percentages and median with P5 and P95 to illustrate ranges. Potential overlap between outcome definitions were visually evaluated using UpSet plots.

Associations between prenatal exposure to acrylamide and the neuropsychiatric disorders were modelled, fitting each outcome separately one-by-one, as incidence rate ratios (IRR) using log linear Poisson models with log follow-up time as offset in the dataset with imputed covariates (N = 861). The Hb adduct levels were fitted as a continuous scale variable (effect estimated per 10-pmol/g Hb increments) or categorized as high (≥P75) vs. low (<P75). The third quartile (P75) was chosen to represent a high acrylamide exposure level while retaining sufficient sample size for comparisons. The third quartile corresponded to 146 pmol/g Hb, which is close to the level of approximately 150 pmol/g Hb above which the exposure-response curves obtained with natural cubic splines deviates from the null (Fig. 1), so that the categorical and the flexible continuous analyses identify the same region of the exposure distribution.

**Fig. 1:**
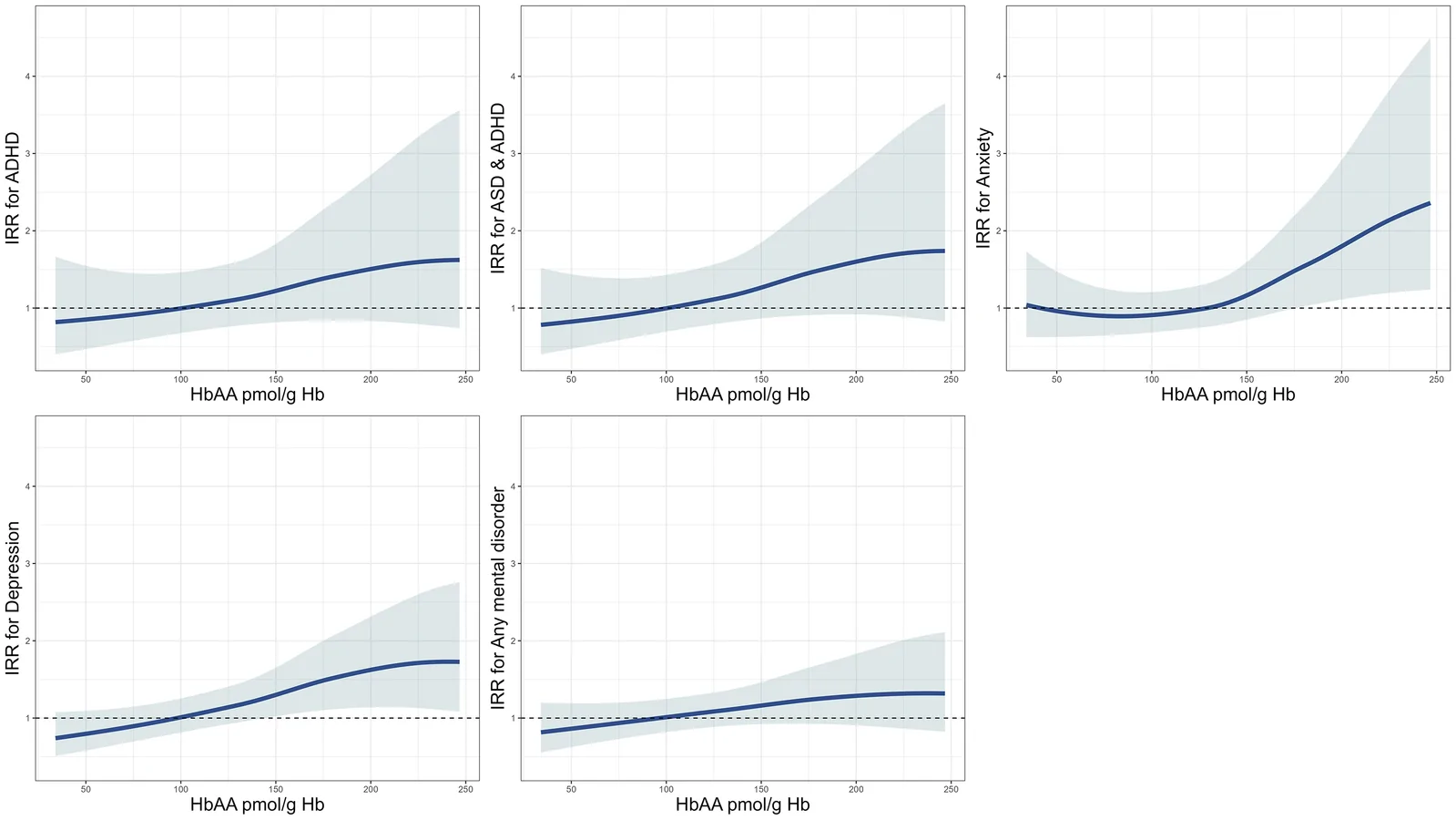
Exposure-response curves of the associations between prenatal exposure to acrylamide and the risk of developing neuropsychiatric disorders. N = 861; Exposure-response curves of the risk of developing anxiety, depression, attention-deficit/hyperactivity disorder (ADHD), co-occurrence of autism (ASD) and ADHD, any neuropsychiatric disorder and HbAA modelled with natural splines with three degrees of freedom; Models were adjusted for maternal smoking, HbEO levels, maternal alcohol intake, maternal healthy eating index, maternal education, household income, parity, sex, and offspring’s gestational age; Likelihood ratio test of the models with HbAA modelled as linear term and with natural splines were statistically significantly for anxiety (p < 0.001) and depression (p < 0.05).

We reported statistical significance with IRR and their corresponding 95% CIs for the most comprehensive adjusted models.

The exposure–response relationships were furthermore assessed using natural cubic splines with three degrees of freedom for continuous Hb adduct levels. In addition, HbAA was categorised into quartiles, with the lowest quartile as the reference, and a test for trend was obtained.

Since tobacco smoking is a source of acrylamide, we furthermore evaluate individual and combined effects of prenatal exposure to acrylamide and maternal smoking on the outcomes.

We used multiple imputations by chained equations (MICE) with the mice R package. The percentage of missing values for individual covariates ranged from 2.6% (for HEI) to 7.8% (for HbEO) resulting in participants having missing information on one or more covariates being 20.2% (n = 174). Analyses were performed on 40 imputed datasets with 25 iterations each to improve imputation quality, stabilize and reflect realistic distributions. Results of each imputations were pooled according to Rubin’s rules to account for the uncertainty inherent in the imputation process.^36^ The assumption that covariate data were missing at random cannot be verified. Participants with and without missing covariate information were comparable with respect to the measured characteristics as shown in Supplementary Table S2.

To assess the robustness of our findings, we performed several sensitivity analyses. In short, we conducted incremental adjustment models; stratification by maternal smoking; Cox models with offspring age as the timescale; restriction to complete cases; different imputation methods; restriction to term births; exclusion of offspring that was small-for-gestational age at birth^13^ and restriction to the HbGA subset to compare results for both biomarkers. We furthermore excluded the three participants whose blood sample was collected in 37 gestational weeks.

Lastly, we tested potential effect modification by maternal smoking, maternal education, any maternal neuropsychiatric diagnosis (both prenatal and postnatal) and sex by adding interaction terms between HbAA as continuous term and these covariates in the further adjusted models.

All the analyses were conducted in R with Rstudio (RStudio Team, 2020) and the packages “Surv” and “glm” at the secure servers of Denmark Statistics.

### Role of the funding source

The funder of the study had no role in study design, data collection, data analysis, data interpretation, or writing of the report.

## Results

### Study population characteristics

Participants inclusion and final sample size is shown in Supplementary Fig. S2. Participating mothers were mostly of high education (54%) and nonsmokers (58%; Table 1). The median level (P5–P95) of HbAA was 93.0 (45.0–249.7) pmol/g Hb (Supplementary Fig. S3). Offspring were more often males (53%), first born (59%), and rarely preterm (3%). By the end of follow-up 34 years after birth, 22% of the offspring had received one or more registrations of any neuropsychiatric diagnosis, 21% of depression, 11% of anxiety, 8% of co-occurrence of ASD and ADHD, and 7% of ADHD cases. Overlap between these neuropsychiatric disorders is presented in Supplementary Fig. S4.

**Table 1:** Study population characteristics for all and by low and high exposure to acrylamide.

Variable	All (n = 687) P50 (P5–P95)/n (%)	Low HbAA < P75 (n = 517) P50 (P5–P95)/n (%)	High HbAA ≥ P75 (n = 170) P50 (P5–P95)/n (%)
Maternal education^a^			
Low	64 (9.3%)	40 (7.7%)	24 (14.1%)
Middle	252 (36.7%)	170 (32.9%)	82 (48.2%)
High	371 (54.0%)	307 (59.4%)	64 (37.7%)
Maternal income (DKK/year)^a^			
≤75,000	150 (21.8%)	106 (20.5%)	44 (25.9%)
75,000–95,000	177 (25.8%)	138 (26.7%)	39 (22.9%)
95,000–115,000	186 (27.1%)	133 (25.7%)	53 (31.2%)
>115,000	174 (25.3%)	140 (27.1%)	34 (20%)
Any maternal neuropsychiatric disorder^b^	89 (12.9%)	58 (11.2%)	31 (18.2%)
Maternal healthy eating index (1–80)^c^	34.4 (23.0–46.0)	35.2 (23.0–46.2)	32.2 (22.9–43.7)
Maternal alcohol intake (g/day)^c^	1.7 (0.0–10.7)	1.7 (0.0–8.5)	2.0 (0.0–13.7)
Maternal active smoking^c^	288 (41.9%)	141 (27.3%)	147 (86.5%)
HbEO (pmol/g Hb)^d^	87.0 (35.0–437.8)	71.0 (33.0–236.4)	268.5 (46.4–660.5)
HbAA (pmol/g Hb)^d^	93.0 (45.0–249.7)	77.0 (42.8–135.2)	196.5 (151.0–318.6)
HbGA (pmol/g Hb)^d^	150.5 (54–404.9)	95.0 (48.8–210.0)	232.0 (121.5–467.5)
Male sex	366 (53.3%)	281 (54.4%)	85 (50%)
Birth weight (g)	3520 (2630–4328)	3587 (2727–4417)	3301 (2526–4016)
Small for gestational age	33 (4.8%)	17 (3.3%)	16 (9.4%)
Gestational age at birth (days)	284 (266–297)	284.0 (266.8–297.0)	283.0 (266.0–295.6)
Preterm	19 (2.8%)	13 (2.5%)	6 (3.5%)
First born child	406 (59.1%)	312 (60.3%)	94 (55.3%)
Depression	146 (21.3%)	100 (19.3%)	46 (27.0%)
Age at first record (year), cases	22.4 (15.2–32.5)	22.5 (15.2–33.0)	22.3 (15.2–32.5)
Anxiety	72 (10.5%)	44 (8.5%)	28 (16.5%)
Age at first record (year), cases	22.8 (11.9–30.8)	23.2 (12.4–31.2)	21.6 (10.1–29.2)
ADHD	45 (6.6%)	29 (5.6%)	16 (9.4%)
Age at first record (year), cases	22.7 (7.3–32.2)	22.9 (7.5–31.4)	22.7 (8.5–33.2)
Co-occurrence of ASD and ADHD	53 (7.7%)	35 (6.8%)	18 (16.6%)
Age at first record (year), cases	21.7 (6.5–32.1)	21.7 (6.5–31.2)	21.6 (8.9–33.2)
Any neuropsychiatric disorder^d^	150 (21.8%)	105 (20.3%)	45 (26.5%)
Age at first record (year), cases	20.2 (7.0–32.0)	20.3 (7.3–31.9)	19.9 (6.8–32.3)

P50, P5, P95 refers to the 50th, 5th, and 95th percentiles; n is the number of observations.

a Year of delivery.

b Diagnosis recorded before and after delivery.

c During pregnancy.

d Gestational week 30; HbAA: Haemoglobin adducts of acrylamide; HbGA: Haemoglobin adducts of glycidamide; HbEO: Haemoglobin adducts of ethylene oxide; ADHD: attention-deficit/hyperactivity disorder; ASD: autism spectrum disorder. All cases are based on ICD8 and ICD10 diagnosis; cases of depression, anxiety, attention-deficit/hyperactivity disorder (ADHD), and co-existing autism spectrum disorder and ADHD additionally include prescribed medication codes. The percentage of missing observations are 5.1% for education, 1.7% for income, 6.4% for smoking, 7.8% for HbEO, 56.3% for HbGA, and 7.3% for gestational age.

Women who smoked during pregnancy had substantially higher HbAA levels as compared to non-smoking women (148.0 vs. 74.0 pmol/g Hb) (Supplementary Table S3). Similarly, the median levels of HbGA and HbEO were also highest for smokers. The women who smoked and women with high HbAA levels were more commonly of low education, low income, and had more often received a neuropsychiatric diagnosis (Supplementary Table S3). Anxiety and ADHD were more common among offsprings of smokers and among those with the highest HbAA levels (Supplementary Table S3). There were 87% smokers in the high acrylamide group and 27% in the low exposed group (Table 1).

### Prenatal exposure to acrylamide and neuropsychiatric disorders

As shown in Table 2, prenatal exposure to acrylamide, measured as HbAA, was associated with IRRs above one for all the outcomes evaluated. When HbAA was linearly modelled per 10-pmol/g Hb increases, none of these associations reached statistical significance (Table 2). Each 10-pmol/g Hb increase in HbAA was associated with an adjusted risk estimate (IRR) of 1.02 (95% CI: 0.99, 1.05) and 1.03 (95% CI: 0.99, 1.07) for depression and anxiety, respectively. The effect estimates were 1.64 (1.05, 2.56) for depression and 2.28 (1.22, 4.26) for anxiety when comparing high with low level (Table 2). The incidence rate of depression was 6.6 per 1000 person-years among the offspring with low prenatal exposure and 9.2 per 1000 person-years among those with high prenatal exposure, and the corresponding rates for anxiety were 2.7 and 5.3 per 1000 person-years.

**Table 2:** Adjusted associations between prenatal exposure to acrylamide and the risk of developing neuropsychiatric disorders in the first 34 years of life (N = 861).

Neuropsychiatric disorders (cases)	Incidence rate per 1000 person-year	Acrylamide per 10- pmol/g Hb increments IRR (95% CI)	High Acrylamide (≥P75 vs. <P75) IRR (95% CI)
Depression (N = 146)	<P75 = 6.7; ≥P75 = 9.3	1.02 (0.99, 1.05)	1.64 (1.05, 2.56)
Anxiety (N = 72)	<P75 = 2.7; ≥P75 = 5.3	1.03 (0.99, 1.07)	2.29 (1.23, 4.25)
ADHD (N = 45)	<P75 = 1.8; ≥P75 = 2.9	1.02 (0.97, 1.07)	1.56 (0.73, 3.32)
Co-occurrence of ASD and ADHD (N = 53)	<P75 = 2.1; ≥P75 = 3.2	1.02 (0.98, 1.07)	1.49 (0.73, 3.02)
Any neuropsychiatric disorder (N = 150)	<P75 = 6.3; ≥P75 = 9.2	1.01 (0.98, 1.04)	1.41 (0.90, 2.22)

Prenatal exposure to acrylamide modelled as ≥75th percentile HbAA vs. <75th percentile, representing 146 pmol/g Hb; and per 10-pmol/g Hb increments; adjusted model includes maternal active smoking, income, education, parity, sex, offspring’s gestational age, HbEO levels, alcohol intake and healthy eating index. All cases are based on ICD8 and ICD10 diagnosis; cases of depression, anxiety, attention-deficit/hyperactivity disorder (ADHD), and Co-occurrence of autism (ASD) and ADHD additionally include prescribed medication codes.

When HbAA levels were above 150 pmol/g Hb, the risk estimates increased, especially for depression and anxiety, with higher acrylamide exposure (Fig. 1). The same patterns were observed for the other outcomes and for HbGA (Supplementary Fig. S5). Likelihood ratio tests comparing the models with HbAA fitted as a linear term with the models fitted with natural cubic splines rejected linearity for anxiety (p < 0.001) and for depression (p < 0.05). The exposure-response relationships for these two outcomes are therefore not adequately summarised by a single linear slope, which explains why the estimates per 10-pmol/g Hb increment did not reach statistical significance while the comparison of high with low exposure did.

When HbAA was categorised into quartiles, the IRRs for depression were [1.06 (95% CI: 0.68,1.66)] for the second [1.12 (95% CI: 0.70, 1.80)], for the third and [1.78 (95% CI: 1.02, 3.11)] for the fourth quartile compared with the first (p for trend = [0.07]), and the corresponding estimates for anxiety were 0.67 (95% CI: 0.35, 1.29), 0.56 (95% CI: 0.27, 1.17), and 1.35 (95% CI: 0.62, 2.97) (p for trend = [0.80]; Supplementary Table S4).

### Individual and combined effects of prenatal exposure to acrylamide and maternal smoking on the risk of neuropsychiatric disorders

The associations for prenatal exposure to acrylamide were also evident in models without adjustment for maternal smoking (Table 3). While the estimated risk from high vs. low acrylamide exposure increased slightly after adjustment for maternal smoking for depression and anxiety, the risk estimates of acrylamide for other outcomes were weaker in mutually adjusted models. Maternal smoking during pregnancy was associated with an increased risk for all the outcomes (Table 3). The risk associated with AA was higher for ADHD and co-occurrence of ASD and ADHD and these associations attenuated slightly but remained significant for ADHD and co-occurrence of ASD and ADHD after adjustment for HbAA (pmol/g Hb).

**Table 3:** Individual and combined effects of prenatal exposure to acrylamide and maternal active smoking on the adjusted risk of developing neuropsychiatric disorders.

	High Acrylamide (≥P75 vs. <P75) IRR (95% CI)	Maternal smoking (yes vs. no) IRR (95% CI)
Single-exposure models		
Depression (N = 146)	1.62 (1.07, 2.46)	1.11 (0.75, 1.65)
Anxiety (N = 72)	2.26 (1.26, 4.05)	1.36 (0.79, 2.36)
ADHD (N = 45)	1.80 (0.86, 3.74)	2.43 (1.23, 4.80)
Co-occurrence of ASD and ADHD (N = 53)	1.78 (0.89, 3.54)	3.02 (1.62, 5.64)
Any neuropsychiatric disorder (N = 150)	1.46 (0.95, 2.23)	1.40 (0.95, 2.06)
Mutually adjusted-exposure models		
Depression (N = 146)	1.64 (1.05, 2.56)	1.00 (0.66, 1.52)
Anxiety (N = 72)	2.29 (1.23, 4.25)	1.10 (0.60, 2.02)
ADHD (N = 45)	1.56 (0.73, 3.32)	2.29 (1.14, 4.62)
Co-occurrence of ASD and ADHD (N = 53)	1.49 (0.73, 3.02)	2.89 (1.52, 5.48)
Any neuropsychiatric disorder (N = 150)	1.41 (0.90, 2.22)	1.31 (0.87, 1.96)

N = 861 obs; Effect estimates from log linear Poisson regression with high HbAA (≥P75) compared to low HbAA (<P75), representing 146 pmol/g Hb; and maternal smoking yes vs. no.

Adjusted single-exposure models include exposure (either categorical variable for HbAA (≥P75 vs. <P75) or maternal smoking (yes vs. no)) adjusted for HbEO levels, maternal alcohol intake, maternal healthy eating index, maternal education, houshold income, parity, sex and offspring’s gestational age. Mutually adjusted models include both exposures simultaneously (both categorical variable for HbAA (≥P75 vs. <P75) and maternal smoking (yes vs. no) adjusted for HbEO levels, maternal alcohol intake, maternal healthy eating index, maternal education, household income, parity, sex and offspring’s gestational age.

All cases are based on ICD8 and ICD10 diagnosis; cases of depression, anxiety, attention-deficit/hyperactivity disorder (ADHD), and co-occurrence of autism (ASD) and ADHD additionally include prescribed medication codes.

### Sensitivity analyses

Although with wide 95% CI, the point estimates showed increased risks from acrylamide exposure across all outcomes in all models with different degrees of adjustment with exception of co-occurrence of ASD and ADHD in the basic adjusted model (adjusted for maternal smoking, income and education; Supplementary Table S5).

The increased risks from high acrylamide exposure vs. low were observed in both offspring of nonsmokers and smokers (Supplementary Table S5). For instance, the risk for depression was 1.79 (95% CI: 0.58, 3.48) in offspring of nonsmokers and 1.47 (95% CI: 0.82, 2.63) in offspring of smokers.

The results presented in Table 2 were robust across various adjustment strategies and modelling approaches (Supplementary Tables S5 and S6). Sensitivity analyses showed robust, directionally consistent associations after different methods of imputation, excluding preterm births, small-for-gestational age offspring or those with maternal neuropsychiatric diagnoses, with only minor attenuation (Supplementary Table S7). Associations weakened in the smaller HbGA subset (n = 363) with wider confidence intervals, though HbGA patterns remained consistent with HbAA (Supplementary Tables S7 and S8; Supplementary Fig. S5). Excluding the three participants whose blood sample was collected in gestational week 37 left the estimates for HbAA in continuous scale virtually unchanged and slightly attenuated the estimates for HbAA dichotomised (Supplementary Table S7).

There was no significant evidence of effect modification (p_interaction_<0.05) by maternal smoking, education, mental health or sex on the associations between prenatal exposure to acrylamide and the neuropsychiatric disorders studied (Supplementary Table S9).

## Discussion

This prospective birth cohort study with 34 years of follow-up of the mental health of the offspring provides longitudinal evidence that prenatal exposure to acrylamide, independent of maternal smoking, is associated with significantly increased risks of onset of depression and anxiety in the childhood and young adulthood. Prenatal exposure to acrylamide was also associated with non-significant increased risk for ADHD, the co-occurrence of ADHD and ASD, and any neuropsychiatric diagnosis.

In line with experimental and occupational evidence of neurotoxicity,^20^, ^21^, ^22^, ^23^, ^26^, ^37^ sparse evidence relating neuropsychiatric disorders associated with long-term low exposure to acrylamide in the general population where the main sources are diet and tobacco smoke has emerged. Specifically, two cross-sectional studies from the US that reported associations between higher levels of HbAA and increased odds of depression symptoms in adults,^28^, ^29^ and one cross-sectional study with children from the US that suggested a link between childhood exposure to the acrylamide metabolite, glycidamide, and increased odds of parent-reported developmental disabilities.^30^ These studies did not evaluate prenatal exposure and relied on questionnaire-based or parent-reported symptom measures, which, as reflected in the little available literature, capture some dimensions of mental health, but may not reflect clinically diagnosed neuropsychiatric disorders. Nevertheless, and consistent with these studies, we observed that prenatal exposure to acrylamide was associated with subsequent depression and anxiety in adolescents and adults.

Our study expanded the evidence by focusing on an early and critically vulnerable exposure window (e.g., prenatal), by using clinically ascertained measures (e.g., diagnosis and medication) with a clear temporal separation and sequence between the exposure to acrylamide, quantified as HbAA in prenatal life, and the subsequent disorder onset.

We also addressed the limitations of previous studies by exploring whether the observed associations for acrylamide were independent of maternal smoking and whether acrylamide mediated the association between maternal smoking during pregnancy and offspring depression, by modelling both exposures independently as well as concurrently. We observed that HbAA levels were associated with increased risk in the full study population and after restriction to nonsmokers. These findings indicated that HbAA may be an independent risk factor for depression and anxiety when controlling maternal smoking and other confounders, suggesting that prenatal exposure to acrylamide both in the full study population as well as in the subset of offspring of nonsmokers and of mothers with acrylamide-rich diet face increased risk of developing depression and anxiety.

Our effect estimate for depression associated with high acrylamide exposure (IRR 1.64, 95% CI 1.05–2.56) was larger than those reported from cross-sectional studies,^28^, ^29^, ^30^ in which the highest vs. the lowest HbAA category was associated with a relative increase in odds of [2.47 (95% CI 1.29–4.77)]^29^ and [1.14 (1.05–1.24)]^28^ for depressive symptoms. In children, glycidamide adducts levels were associated with an odds ratio of [2.16 (1.27–3.68)] for parent-reported developmental disabilities,^30^ and dietary acrylamide intake was associated with an odds ratio of [3.4 (1.06–10.59)] for mild cognitive decline in elderly nonsmoking men.^24^ Several differences may explain why our estimates are larger; the cross-sectional studies were limited by measuring symptoms and proxy-measures rather than clinically diagnoses. Other study design related differences may also partly explain the difference, for instance our study population was more homogen than those of the cross-sectional studies from the US, we could also control for a wider range of covariates and the follow-up time was longer in our study. It could also be that the difference reflect that prenatal exposure compared to exposure later in life such as during mid adulthood has a stronger basis for the neurotoxic effects contributing to the development of neuropsychiatric disorders. In our study, the observed associations between HbGA and neuropsychiatric disorders, although directionally consistent with those from HbAA, are more uncertain (wide 95% CI) given these analyses were conducted in a subsample of participants with quantified glycidamide (n = 363). Biological plausibility is supported by extensive animal and *in vitro* studies demonstrating that acrylamide causes neurotoxicity and developmental neurotoxicity.^20^ Although mechanisms are incompletely understood, they may involve axonal degeneration, neuronal apoptosis and autophagy, oxidative stress with reduced antioxidant defences, neuroinflammation, mitochondrial dysfunction,^38^, ^39^, ^40^, ^41^ disrupted neuronal migration and differentiation, and impaired axonal transport machinery.^42^, ^43^, ^44^ Noteworthy, a human neuroblastoma cell study has reported that acrylamide at low concentrations attenuated neural differentiation and dysregulated mRNA markers for neural development, however the neuronal differentiation was not linked with GSH depletion or induction of oxidative stress, suggesting that other mechanisms are involved.^45^

Rodent studies show that gestational exposure to acrylamide causes structural brain changes in offspring, including altered hippocampal development, reduced neurotrophic factor expression, and persistent motor and cognitive deficits into adulthood.^46^, ^47^, ^48^ These studies indicate a change in the brain structure that may translate into long-term changes in behaviour, suggesting that prenatal exposure to acrylamide may disrupt critical time windows of neurodevelopmental processes that extend beyond early life into adulthood. Longitudinal animal studies have linked chronic exposure to acrylamide with anxiety, depressive-like behaviour, and cognitive decline, potentially mediated by neuroinflammation and disrupted lipid metabolism.^49^

A major strength of our study was that it fills critical knowledge gap as no previous studies have evaluated effects of prenatal exposure to acrylamide and it responds to previous calls^20^, ^21^, ^28^, ^29^ for a longitudinal prospective study design that establishes the temporal sequence between prenatal exposure to acrylamide and onset of neuropsychiatric disorders later in life, eliminating the possibility of reverse causality. Additional strengths include the ability to quantify Hb adducts from acrylamide and ethylene oxide in maternal blood collected during a critical window of prenatal development and growth as part of a well-characterised birth cohort; to adjust for a wider range of confounders; and to link with high-quality registries allowing up to 34 years of follow-up of the clinical defined neuropsychiatric disorders of the full population. Neuropsychiatric disorders were captured using both clinical diagnostic codes and medication records made by professionals using international clinical criteria which enabled us to evaluate specific subtypes of disorders and to capture a wide range of cases which minimise the risk of measurement error, recall bias or selection bias. Residents in Denmark have free access to health care which reduce residual confounding by socio-economic factors. Our development and application of the high throughput quantification of Hb adducts in a large number of samples^34^, ^35^ enabled a more accurate assessment of the individual exposure to dietary acrylamide and tobacco smoke than if we had relied on self-reported questionnaire-based data solely. The concentrations of HbAA reflect maternal internal dose of acrylamide from all sources and together with the HbEO and the detailed individual level data on offspring health and maternal smoking, dietary habits and other characteristics collected during pregnancy contributed to the strengths of our study. With this data we have previously reported that higher HbAA levels were associated with restricted fetal growth^13^ and overall, no compelling evidence for an association with offspring metabolic health at 20 years of age.^35^

A limitation of the study was the relatively small population size, restricting statistical power to study more rare disorders relative to depression such as ASD for which only 11 offspring were identified in our study. The number of cases was relatively low for ADHD (n = 45) and for the co-occurrence of ADHD and ASD (n = 53), and the corresponding 95% CIS related to analyses of these were wide for models fitted with the dichotomised exposure variable. The estimates for these outcomes were underpowered and did not reach statistical significance; therefore, they should not be interpreted as evidence of the absence of an effect. With 45 cases and approximately one quarter of the population classified as highly exposed, our study had 80% power to detect an IRR of 2.6 for ADHD and of 2.4 for the co-occurrence of ADHD and ASD. Complementary, detecting the observed associations with 80% power would have required approximately 4050 participants for ADHD and 4280 participants for the co-occurrence of ASD and ADHD, assuming the same proportion highly exposed and event rates. The study population size also prevented the possibility to study subtypes of ASD, certain outcomes such as schizophrenia, bipolar disorders, etc. In order to evaluate associations between acrylamide and these rarer disorders, we grouped multiple diagnosis under any neuropsychiatric disorder(s), which represent a heterogeneous group of pathology with distinct mechanisms and as a composite estimate that should be interpreted with caution. We combined diagnosis with medicine prescriptions in order to capture all cases, but it might also have resulted in creation of a heterogeneous group of different severities and pathology. Some misclassification may exist when identifying cases of depression and anxiety using medications, as some of these may overlap. Diagnosis from both psychiatric and nonpsychiatric public departments were captured from inpatients, outpatients and emergency rooms for the period of relevance for our study as well as from private hospitals and private specialty clinics from 2003 onwards,^50^ but we may have lack cases not diagnosed or only diagnosed before 2003 on a private hospital/clinic for those not prescribing medication.

Furthermore, another limitation was that HbAA levels were determined in samples collected a onetime point only for the majorities of the participants. Measuring exposure over a single period during prenatal development and having a long follow-up period, may not fully capture later changes in maternal and offspring acrylamide-related dietary or lifestyle habits, such as smoking. Our estimates should therefore be interpreted as associations with prospectively measured prenatal exposure, acknowledging that subsequent exposure may contribute to the observed associations. Because dietary habits tend to be similar within families, with offspring dietary habits at 14 years of age being highly correlated with maternal dietary habits during pregnancy,^49^ prenatal exposure may also capture, in part, patterns of exposure that persist across childhood and later life. However, because prenatal development and growth represents a biologically sensitive period, studies evaluating associations with prospectively measured prenatal exposure remain informative even when later exposures contribute to the observed effects.^51^

Finally, as this is an observational study we also cannot rule out residual confounding by behavioural factors associated with high acrylamide exposure, or by maternal mental health, despite extensive adjustments and modelling. Given the imbalance in smoking prevalence between exposure groups, all models were adjusted for HbEO on a continuous scale, as internal dose biomarker for exposure to tobacco smoke (active and passive smoking), residual confounding by the intensity and the duration of smoking during pregnancy cannot be excluded. Active smoking during pregnancy was common in our study population (42%), which reflects a cultural norm among pregnant women in Denmark in 1988–1989, and is considerably higher than today. Overall, our findings are consistent with a contribution of early-life exposure to ubiquitous neurotoxicants to the developmental origins of neuropsychiatric disorders, but they cannot on their own establish that prenatal exposure is the responsible window, and they require replication in cohorts with repeated exposure measurements. The findings of our study support an association between prenatal exposure to acrylamide and certain subsequent neuropsychiatric outcomes, but they do not establish a causal effect of prenatal exposure or demonstrate that an intervention reducing prenatal exposure to acrylamide would reduce the incidence of these disorders.

Five outcomes were examined, and the reported confidence intervals and p-values were not adjusted for multiple comparisons. Although depression and anxiety were defined as the primary outcomes a priori, and the outcomes overlap substantially, the possibility of chance findings cannot be excluded, with these associations requiring replication in independent populations.

The study population, complemented with the high coverage of the Danish register data, was recruited at a single hospital and was relatively homogeneous, well-resourced and of Danish origin, which reduces confounding by socioeconomic factors, but limits generalisation to more diverse populations.

In conclusion, prenatal exposure to acrylamide was associated with a 1.6-fold higher incidence of depression and a 2.3-fold higher incidence of anxiety in the offspring, independently of maternal active smoking during pregnancy and potential confounders evaluated. The estimates for ADHD, for the co-occurrence of ADHD and ASD and for any neuropsychiatric diagnosis pointed in the same increasing direction, but were underpowered and associations did not reached statistical significance. If confirmed, given that exposure to acrylamide is ubiquitous, and the large burden of depression and anxiety, these findings support continued efforts to decrease exposure to acrylamide from both diet and tobacco smoke to pregnant women.

## Contributors

DCP: Data curation, Formal analysis, Software, Investigation, Visualisation, Writing–original draft. ST: Data curation, Formal analysis, Software, Investigation, Visualisation, Writing–reviewing & editing. EV: Data curation, Software, Investigation, Writing–reviewing & editing. TIH: Data curation, Resources, Investigation, Writing–reviewing & editing. AAB: Writing–reviewing & editing. MS: Writing–reviewing & editing. DR: Data curation, Methodology, Writing–reviewing & editing. BHB: Data curation, Methodology, Writing–reviewing & editing. TBH: Data curation, Methodology, Writing–reviewing & editing. SFO: Data curation, Resources, Investigation, Methodology, Writing–reviewing & editing. MT: Conceptualisation, Funding acquisition, Resources, Data curation, Validation, Investigation, Writing–reviewing & editing. MP: Conceptualisation, Funding acquisition, Resources, Investigation, Methodology, Project administration, Supervision, Writing–original draft & editing. DCP & ST: Accessed and verified the data. All authors read and approved the final version of the manuscript.

## Data sharing statement

The data are protected by personal data legislation and cannot be shared.

## Declaration of interests

MP has received funding in 2017 for this study from the European Research Council (ERC) under the European Union’s Horizon 2020 research and innovation programme (grant agreement No 758151). MP has received consulting fees from Danish Council of Prevention for writing a report on the health effects of exposure to air pollution and actions for reducing air pollution. MP, BHB and MT own stock or stock options privately, but none of the companies are related to acrylamide or foods. MP is a board member of the non-governmental organization ‘Green Global Future’. MT has received funding as a collaborator in 2018 for this specific study from the European Research Council (ERC) under the European Union’s Horizon 2020 research and innovation programme (grant agreement No 758151). MT has also received support for research infrastructure, salaries and co-financing of OH costs from Stockholm University. SFO has received research support from the Innovation Fund Denmark and Danish Council for Strategic Research in 2010 (grant no 09-075611, earlier grant no 09-067124), which enabled the undertaking of the present study. Other authors have no conflict of interest to disclose.

## References

[1] Tareke E., Rydberg P., Karlsson P., Eriksson S., Törnqvist M. (2002). Analysis of acrylamide, a carcinogen formed in heated foodstuffs. J Agric Food Chem.

[2] Mottram D.S., Wedzicha B.L., Dodson A.T. (2002). Acrylamide is formed in the Maillard reaction. Nature.

[3] European Food Safety Authority (EFSA) (2015). Panel on contaminants in the Food Chain (CONTAM). Scientific opinion on acrylamide in food. EFSA J.

[4] Hoseini Majd S., Shahidi S.A., Shariatifar N., Ahmadi M., Sharifi Soltani M. (2025). Acrylamide occurrence in Iranian biscuits and its potential risk of exposure. Food Sci Nutr.

[5] Shariatifar N., Arabameri M., Asghari H. (2026). Measurement of acrylamide in traditional breads sold in Tehran: a health risk assessment study. Iran J Health Environ.

[6] Abt E., Robin L.P., McGrath S. (2019). Acrylamide levels and dietary exposure from foods in the United States, an update based on 2011-2015 data. Food Addit Contam Part A.

[7] Timmermann C.A.G., Mølck S.S., Kadawathagedara M. (2021). A review of dietary intake of acrylamide in humans. Toxics.

[8] Pedersen M., Vryonidis E., Joensen A., Törnqvist M. (2022). Hemoglobin adducts of acrylamide in human blood – what has been done and what is next?. Food Chem Toxicol.

[9] Vikström A.C., Warholm M., Paulsson B., Axmon A., Wirfält E., Törnqvist M. (2012). Hemoglobin adducts as a measure of variations in exposure to acrylamide in food and comparison to questionnaire data. Food Chem Toxicol.

[10] International Agency for Research on Cancer (IARC) (1994). IARC monographs on the evaluation of carcinogenic risks to humans.

[11] Annola K., Keski-Rahkonen P., Vähäkangas K., Lehtonen M. (2008). Simultaneous determination of acrylamide, its metabolite glycidamide and antipyrine in human placental perfusion fluid and placental tissue by liquid chromatography–electrospray tandem mass spectrometry. J Chromatogr B.

[12] Vrachnis N., Loukas N., Antonakopoulos N. (2023). Detection and quantification of acrylamide in second trimester amniotic fluid using a novel LC-MS/MS technique to determine whether high acrylamide content during pregnancy is associated with fetal growth. Biology.

[13] Chandia-Poblete D., Tuffier S., Vryonidis E. (2025). Prenatal exposure to acrylamide, fetal growth and newborn size: a biomarker-based cohort study from Denmark. Environ Res.

[14] Hogervorst J., Virgolino A., Halldorsson T.I. (2022). Maternal acrylamide exposure during pregnancy and fetal growth: a systematic review and dose-response meta-analysis of epidemiological studies. Environ Res.

[15] Pedersen M., von Stedingk H., Botsivali M. (2012). Birth weight, head circumference, and prenatal exposure to acrylamide from maternal diet: the European prospective mother-child study (NewGeneris). Environ Health Perspect.

[16] Freire M.B.O., Slater R., Santos T.M., da Silva B.G.C., Baxter L., Menezes A.M.B. (2024). Head circumference and intelligence, schooling, employment, and income: a systematic review. BMC Pediatr.

[17] Zhu Z., Shen J., Zhu Y. (2022). Head circumference trajectories during the first two years of life and cognitive development, emotional, and behavior problems in adolescence: a cohort study. Eur J Pediatr.

[18] von Stedingk H., Vikstrom A.C., Rydberg P. (2011). Analysis of hemoglobin adducts from acrylamide, glycidamide, and ethylene oxide in paired mother/cord blood samples from Denmark. Chem Res Toxicol.

[19] Álvarez-Mejía D., Rodas J.A., Leon-Rojas J.E. (2025). From womb to mind: prenatal epigenetic influences on mental health disorders. Int J Mol Sci.

[20] Lindeman B., Johansson Y., Andreassen M. (2021). Does the food processing contaminant acrylamide cause developmental neurotoxicity? A review and identification of knowledge gaps. Reprod Toxicol.

[21] Matoso V., Bargi-Souza P., Ivanski F., Romano M.A., Romano R.M. (2019). Acrylamide: a review about its toxic effects in the light of Developmental Origin of Health and Disease (DOHaD) concept. Food Chem.

[22] Godin A.C., Bengtsson B., Niskanen R., Tareke E., Törnqvist M., Forslund K. (2002). Acrylamide and N-methylolacrylamide poisoning in a herd of Charolais crossbreed cattle. Vet Rec.

[23] Hagmar L., Törnqvist M., Nordander C. (2001). Health effects of occupational exposure to acrylamide using hemoglobin adducts as biomarkers of internal dose. Scand J Work Environ Health.

[24] Liu Z.M., Tse L.A., Chen B. (2017). Dietary acrylamide exposure was associated with mild cognition decline among non-smoking Chinese elderly men. Sci Rep.

[25] Mulloy K.B. (1996). Two case reports of neurological disease in coal mine preparation plant workers. Am J Ind Med.

[26] National Toxicology Program (NTP) (2012). Toxicology and carcinogenesis studies of acrylamide (CAS No. 79-06-1) in F344/N rats and B6C3F1 mice (feed and drinking water studies). Natl Toxicol Program Tech Rep Ser.

[27] Calleman C.J. (1996). The metabolism and pharmacokinetics of acrylamide: implications for mechanisms of toxicity and human risk estimation. Drug Metab Rev.

[28] Guo J., Garshick E., Si F. (2024). Environmental toxicant exposure and depressive symptoms. JAMA Netw Open.

[29] Li Z., Sun J., Zhang D. (2021). Association between acrylamide hemoglobin adduct levels and depressive symptoms in US adults: NHANES 2013–2016. J Agric Food Chem.

[30] Meng F., Qi Y., Wu Y., He F. (2022). Association between acrylamide exposure and the odds of developmental disabilities in children: a cross-sectional study. Front Public Health.

[31] Olsen S., Hansen H.S., Secher N.J., Jensen B., Sandström B. (1995). Gestation length and birth weight in relation to intake of marine n-3 fatty acids. Br J Nutr.

[32] Schmidt M., Pedersen L., Sørensen H.T. (2014). The Danish Civil Registration System as a tool in epidemiology. Eur J Epidemiol.

[33] von Stedingk H., Rydberg P., Törnqvist M. (2010). A new modified Edman procedure for analysis of N-terminal valine adducts in hemoglobin by LC–MS/MS. J Chromatogr B.

[34] Vryonidis E., Törnqvist M., Lignell S., Rosén J., Aasa J. (2024). Estimation of intake and quantification of hemoglobin adducts of acrylamide in adolescents in Sweden. Front Nutr.

[35] Tuffier S., Vryonidis E., Halldorsson T.I. (2025). Prenatal exposure to acrylamide and metabolic health at 20 years of age A biomarker-based Danish cohort study. Environ Int.

[36] van Buuren S., Groothuis-Oudshoorn K. (2011). mice: multivariate imputation by chained equations in R. J Stat Softw.

[37] Calleman C., Wu Y., He F. (1994). Relationships between biomarkers of exposure and neurological effects in a group of workers exposed to acrylamide. Toxicol Appl Pharmacol.

[38] Zhao M., Zhang B., Deng L. (2022). The mechanism of acrylamide-induced neurotoxicity: current status and future perspectives. Front Nutr.

[39] Baraka S.M., Hussien Y.A., Ahmed-Farid O.A., Hassan A., Saleh D.O. (2026). Neuroprotective potential of eugenol against acrylamide-induced brain toxicity by regulating Nrf2/NQO1/HO-1 and NLRP3/NF-κB/IL-1β signaling cascades. Sci Rep.

[40] Zhang L., Yang L., Luo Y., Dong L., Chen F. (2023). Acrylamide-induced hepatotoxicity through oxidative stress: mechanisms and interventions. Antioxid Redox Signal.

[41] Allam A., El-Ghareeb A., Abdul-Hamid M., Baikry A., Sabri M. (2011). Prenatal and perinatal acrylamide disrupts the development of cerebellum in rat: biochemical and morphological studies. Toxicol Ind Health.

[42] Attoff K., Johansson Y., Cediel-Ulloa A. (2020). Acrylamide alters CREB and retinoic acid signalling pathways during differentiation of the human neuroblastoma SH-SY5Y cell line. Sci Rep.

[43] Gold B.G., Griffin J.W., Price D. (1985). Slow axonal transport in acrylamide neuropathy: different abnormalities produced by single-dose and continuous administration. J Neurosci.

[44] LoPachin R.M., Gavin T. (2012). Molecular mechanism of acrylamide neurotoxicity: lessons learned from organic chemistry. Environ Health Perspect.

[45] Johansson Y., Andreassen M., Hartsch M., Wagner S., Forsby A. (2024). Attenuated neuronal differentiation caused by acrylamide is not related to oxidative stress in differentiated human neuroblastoma SH-SY5Y cells. Food Chem Toxicol.

[46] Erdemli M., Turkoz Y., Altinoz E., Elibol E., Dogan Z. (2016). Investigation of the effects of acrylamide applied during pregnancy on fetal brain development in rats and protective role of the vitamin E. Hum Exp Toxicol.

[47] Liu Y., Yan D., Wang Y. (2021). Subchronic exposure to acrylamide caused behaviour disorders and related pathological and molecular changes in rat cerebellum. Toxicol Lett.

[48] Liu Y., Wang Y., Zhang X. (2022). Chronic acrylamide exposure resulted in dopaminergic neuron loss, neuroinflammation and motor impairment in rats. Toxicol Appl Pharmacol.

[49] Quan W., Li M., Jiao Y. (2022). Effect of dietary exposure to acrylamide on diabetes-associated cognitive dysfunction from the perspectives of oxidative damage, neuroinflammation, and metabolic disorders. J Agric Food Chem.

[50] Plana-Ripoll O., Liu X., Köhler-Forsberg O., Sørensen H.T., Momen N.C. (2025). Mental disorders in Danish Hospital Registers: a review of content and possibilities for epidemiological research. Clin Epidemiol.

[51] Ahrendt Bjerregaard A., Halldorsson T.I., Tetens I., Frodi Olsen S. (2019). Mother’s dietary quality during pregnancy and offspring’s dietary quality in adolescence: follow-up from a national birth cohort study of 19,582 mother–offspring pairs. PLoS Med.

