# Supplementary material for: Prenatal exposure to acrylamide and neuropsychiatric disorders: a biomarker-based birth cohort study with 34 years of follow-up
Source: eClinicalMedicine. 2026 Oct 26;101:104286. doi: 10.1016/j.eclinm.2026.104286 (PMC13734943; doi:10.1016/j.eclinm.2026.104286)
Supplement: Supplementary file 1 [file mmc1.docx]

**Supplemental Material**

Prenatal exposure to acrylamide and neuropsychiatric disorders: a biomarker-based birth cohort study with 34 years of follow-up

Damian Chandia-Poblete, Stéphane Tuffier, Efstathios Vryonidis, Thorhallur Ingi Halldorsson, Anne Ahrendt Bjerregaard, Marin Strøm, Dorte Rytter, Bodil Hammer Bech, Tine Brink Henriksen, Sjurdur Frodi Olsen, Margareta Törnqvist, Marie Pedersen.

**List of tables and figures**

Table S1. Definition of neuropsychiatric disorders.

Table S2. Study population characteristics for included and excluded participants.

Table S3. Study population characteristics by maternal smoking status.

Table S4. Associations between prenatal exposure to acrylamide modelled as quartiles and the risk of developing neuropsychiatric disorders in the first 34 years of life (N=861).

Table S5. Associations between prenatal exposure to acrylamide and the risk of developing neuropsychiatric disorders in the first 34 years of life in the full study population (N=861), offspring of nonsmokers (n=573) and offspring of women classified as active smokers during pregnancy (n=288).

Table S6. Hazard ratios for the associations between prenatal exposure to acrylamide and the risk of developing neuropsychiatric disorders in the first 34 years of life in the full study population (N=861), offspring of nonsmokers (n=573) and offspring of women classified as active smokers during pregnancy (n=288).

Table S7. Associations between prenatal exposure to acrylamide and mental health in the first 34 years of life - sensitivity analyses.

Figure S1. DAG of confounders in prenatal acrylamide and neuropsychiatric disorders

Figure S2. Flow-chart of the number of participants and reasons for attrition

Figure S3. Upset plot of the relationship among neuropsychiatric disorders.

Table S8. Associations between prenatal exposure to glycidamide and the risk of developing neuropsychiatric disorders in the first 34 years of life in the full study population (N=363).

Figure S4. Distribution of HbAA (left) and HbGA (right) in the full cohort study population.

Figure S5. Exposure-response curves of the associations between prenatal exposure to glycidamide and the risk of developing neuropsychiatric disorders.

Table S9. Effect modification on the association between prenatal exposure to acrylamide and the risk of developing neuropsychiatric disorders in the first 34 years of life in the full study population (N=861).

**Supplement to Methods section and definition of neuropsychiatric disorders**

*Further details on Study population*

To all women enrolled in the DaFO88 Cohort, a self-administered questionnaire was mailed to them one week before the visit. Following informed consent, women participated in a face-to-face interview and gave information about their diet, lifestyle, and health. We estimated maternal diet quality during pregnancy by calculating the Healthy Eating Index (HEI)^28^.

Using the Danish civil registration number^29^, we linked the cohort data with data on the date of death or emigration from the Danish Civil Registry^30^, household income from the Income Register^29^, maternal education from the Danish Education Registry^31^, parity, sex of the child and gestational age from the medical records and the Danish Medical Birth Registry^32^, diagnosis and date of diagnosis from the Danish National Patient Registry^33^ and the Danish Psychiatric Central Register^34^ to extract data 1988 until 2023; and medications prescribed during 1995 until 2023 from the Danish National Prescription Register^35^.

*Further details on Measurement of haemoglobin adducts biomarkers*

Isolated erythrocytes were stored at –20°C and the adduct levels were measured by the “adduct FIRE procedure” (fluorescein isothiocyanate R Edman) method modified for higher throughput analysis^36^ and applied to studies of offspring foetal growth and metabolic health at 20 years of age^10,37^. In brief, adducted N-terminal valines were detached using fluoresceine-5-isothiocyanate. The detached analytes were purified by solid phase extraction and analysed by liquid chromatography-mass spectrometry^15,36,38^.

*Further details on Sensitivity analyses*

First, we evaluated incremental adjustment models given few observations for some outcomes. Second, we stratified by maternal smoking status to differentiate between dietary and smoking sources of acrylamide. Third, we conductied Cox proportional hazards models with age of the offspring as the underlying timescale, where offspring were followed from birth until diagnosis of a neuropsychiatric disorder or end of follow-up (December 31, 2022), emigration, or death, whichever occurred first. Fourth, we repeated our analysis restricted to complete cases to assess any bias from imputation. Fifth, we tested two additional imputation methodology referred to as S1, which includes 50 imputed datasets with 35 iterations each, and as S2, which includes 15 imputed datasets with 15 iterations each. Sixth, restricted the study population to term births to eliminate potential influence of preterm delivery and we excluded offspring small-for-gestational age at birth to eliminate potential influence of restricted fetal growth and perinatal health. Finally, we restricted the study population to the subset with HbGA available, to compare primary results for both HbAA and HbGA to those of the population with complete biomarker information.

**Table S1. Definitions of neuropsychiatric disorders.**

| **Outcome** | **International Classification of Diseases 8^th^ revision** | **International Classification of Diseases 10^th^ revision** | **Anatomical Therapeutic Chemical codes** | **Case definition** |
| --- | --- | --- | --- | --- |
| Depression | 296·09; 296·29; 298·09;296·99; 298·09;300·49 | F32·0; F32·1; F32·2; F32·3; F32·8; F32·9; F33·0; F33·1; F33·2; F33·3; F33·4; F33·8; F33·9 | ATC: N06A (Antidepressant medication) | The offspring with at least one primary diagnosis, or the offspring who, during follow-up, filled at least two prescriptions |
| Anxiety | 300·0;300·2 | F40-48; F93; | ATC: N05B (Anxiolytic medication) | The offspring with at least one primary diagnosis, or the offspring who, during follow-up, filled at least two prescriptions |
| Attention Deficit Hyperactivity Disorder (ADHD) | 308 | F90; F98·8 | ATC: NO6B (Psychostimulants, agents used for ADHD and nootropics) | The offspring with at least one primary diagnosis, or the offspring who, during follow-up, filled at least two prescriptions |
| Co-occurrence of Autism spectrum disorder (ASD) and ADHD | 299  308 | F84·0; F84·1; F84·5; F84·8; F84·9;  F90; F98·8 | ATC: NO6B (Psychostimulants, agents used for ADHD and nootropics) | The offspring with at least one primary diagnosis, or the offspring who, during follow-up, filled at least two prescriptions |
| Any neuropsychiatric diagnosis | 290-315 | F00-99 | - | The offspring with at least one primary diagnosis of the disorder |

Neuropsychiatric disorders information was obtained through registry linkage (Plana-Ripoll et al, 2025) using the unique Danish civil registration numbers of women and their offspring participating in the DaFO88 cohort (Olsen et al, 1995) Linked registries included the Danish National Patient Register (DNPR, Schmidt et al, 2015), the Danish Psychiatric Central Research Register (PCRR, Mors et al, 2011), and the Registry of Medicinal Products Statistics (RMPS, Pottegård et al, 2017). The DNPR contains records of all inpatient admissions (from 1977), outpatient visits, and emergency room encounters (from 1995), with diagnoses coded using the International Classification of Diseases (ICD) the 8^th^ revision and the 10^th^ revision which was implemented in 1994· It also includes private clinics and hospitals records since 2003. The PCRR provides data on contacts with psychiatric hospitals (from 1969) and outpatient clinics (from 1995). Data on diagnoses from private clinical settings are not available before 2003, thus we used prescription of relevant medication data according to the Anatomical Therapeutic Chemical Classification System (ATC) as indication of neuropsychiatric disorders. The RMPS captures all prescriptions of medications dispensed by Danish pharmacies (from 1995). Inpatient admissions correspond to overnight hospital stays or daily hospital visits over an extended period for diagnostic evaluation and treatment while outpatient admissions correspond to clinic visits on a less regular basis.

**Table S2. Study population characteristics for included and excluded participants.**

|  | **Included (N=687)** | **Excluded (n=295)** |
| --- | --- | --- |
| Variable | P50 (P5-P95) / n (%) | P50 (P5-P95) / n (%) |
| Maternal education ^a^ |  |  |
| Low | 64 (9·3%) | 22 (9·2%) |
| Middle | 252 (36·7%) | 64 (26·7%) |
| High | 371 (54·0%) | 154 (64·2%) |
| Maternal income (DKK/year)^a^ |  |  |
| ≤ 75,000 | 150 (21·8%) | 100 (36·5%) |
| 75,000-95,000 | 177 (25·8%) | 67 (24·5%) |
| 95,000-115,000 | 186 (27·1%) | 49 (17·9%) |
| >115,000 | 174 (25·3%) | 58 (21·2%) |
| Any maternal neuropsychiatric diagnosis ^b^ | 89 (12·9%) | 51 (17·3%) |
| Maternal healthy eating index (1–80) ^c^ | 34·4 (23·0-46·0) | 35·6 (24·5-45·7) |
| Maternal alcohol intake (g) ^c^ | 1·7 (0·0-10·7) | 1·5 (0-9·9) |
| Maternal active smoking ^c^ | 288 (41·9%) | 76 (32·9%) |
| HbEO (pmol/g Hb) ^d^ | 87·0 (35·0-437·8) | 103·5 (37·9-685·5) |
| HbAA (pmol/g Hb) ^d^ | 93·0 (45·0-249·7) | 85 (46-267) |
| HbGA (pmol/g Hb) ^d^ | 150·5 (54-404·9) | 151·5 (52·7-385·5) |
| Male sex | 366 (53·3%) | 110 (49·8%) |
| Birth weight (g) | 3520 (2630-4328) | 3451 (2379-4385) |
| Small for gestational age | 33 (4·8%) | 12 (4·1%) |
| Gestational age at birth (days) | 284 (266-297) | 283 (257·2-298) |
| Preterm | 19 (2·8%) | 20 (7%) |
| First born child | 406 (59·1%) | 87 (29·6%) |
| Depression | 146 (21·3%) | 62 (21·1%) |
| Age at first record (year), cases | 22·4 (15·2-32·5) | - |
| Anxiety | 72 (10·5%) | 26 (8·8%) |
| Age at first record (year), cases | 22·8 (11·9-30·8) | - |
| ADHD | 45 (6·6%) | 14 (4·8%) |
| Age at first record (year), cases | 22·7 (7·3-32·2) | - |
| Co-occurrence of ASD and ADHD | 53 (7·7%) | 16 (5·4%) |
| Age at first record (year), cases | 21·7 (6·5-32·1) | - |
| Any neuropsychiatric diagnosis | 150 (21·8%) | 43 (14·6%) |
| Age at first record (year), cases | 20·2 (7·0-32·0) | - |

^a^ Year of delivery; ^b^ Diagnosis recorded before and after delivery; ^c^ During pregnancy; ^d^ Gestational week 30;

Any maternal neuropsychiatric diagnosis defined as any mother with at least one primary diagnosis of any ICD10: F00-99 code; HbAA: Haemoglobin adducts of acrylamide; HbGA: Haemoglobin adducts of glycidamide; HbEO: Haemoglobin adducts of ethylene oxide; ADHD: attention-deficit/hyperactivity disorder; ASD: autism spectrum disorder. All cases are based on ICD8 and ICD10 diagnosis; cases of depression, anxiety, attention-deficit/hyperactivity disorder (ADHD), and co-existing autism spectrum disorder (ASD) and ADHD additionally include prescribed medication codes. Age at first record contains few missing data and prevented from be reported.

**Table S3. Study population characteristics for all and by maternal smoking status.**

|  | **All (N=687)** | **Nonsmokers (n=399)** | **Smokers (n=288)** |
| --- | --- | --- | --- |
| Variable | P50 (P5-P95) / n (%) | P50 (P5-P95) / n (%) | P50 (P5-P95) / n (%) |
| Maternal education ^a^ |  |  |  |
| Low | 64 (9·3%) | 31 (7·8%) | 33 (11·4%) |
| Middle | 252 (36·7%) | 123 (30·8%) | 129 (44·8%) |
| High | 371 (54·0%) | 245 (61·4%) | 126 (43·8%) |
| Maternal income (DKK/year)^a^ |  |  |  |
| ≤ 75,000 | 150 (21·8%) | 78 (19·5%) | 72 (25·0%) |
| 75,000-95,000 | 177 (25·8%) | 101 (25·3%) | 76 (26·4%) |
| 95,000-115,000 | 186 (27·1%) | 104 (26·1%) | 82 (28·5%) |
| >115,000 | 174 (25·3%) | 116 (29·1%) | 58 (20·1%) |
| Any maternal neuropsychiatric diagnosis ^b^ | 89 (12·9%) | 38 (9·5%) | 51 (17·7%) |
| Maternal healthy eating index (1–80) ^c^ | 34·4 (23·0-46·0) | 35·0 (23·2-46·0) | 33·8 (22·7-46·0) |
| Maternal alcohol intake (g) ^c^ | 1·7 (0·0-10·7) | 1·7 (0·0-8·6) | 1·7 (0·0-11·8) |
| Maternal active smoking ^c^ | 288 (41·9%) | - | - |
| HbEO (pmol/g Hb) ^d^ | 87·0 (35·0-437·8) | 63·0 (33·0-141·1) | 224·5 (55·0-584·2) |
| HbAA (pmol/g Hb) ^d^ | 93·0 (45·0-249·7) | 74·0 (40·9-151·0) | 148·0 (64·0-309·6) |
| HbGA (pmol/g Hb) ^d^ | 150·5 (54-404·9) | 81·0 (41·9-165·1) | 199·0 (81·0-448·4) |
| Male sex | 366 (53·3%) | 216 (54·1%) | 150 (52·1%) |
| Gestational age at birth (days) | 284 (266-297) | 284 (266-298) | 284 (266·4-296) |
| Birth weight (g) | 3520 (2630-4328) | 3580 (2792-4398) | 3416 (2570-4267) |
| Small for gestational age | 33 (4·8%) | 11 (2·8%) | 22 (7·6%) |
| Preterm | 19 (2·8%) | 11 (2·8%) | 8 (2·8%) |
| First born child | 406 (59·1%) | 241 (60·4%) | 165 (57·3%) |
| Depression | 146 (21·3%) | 83 (20·8%) | 63 (21·9%) |
| Age at first record (year), cases | 22·4 (15·2-32·5) | 22·7 (16·1-32·7) | 21·9 (14·6-31·6) |
| Anxiety | 72 (10·5%) | 36 (9·0%) | 36 (12·5%) |
| Age at first record (year), cases | 22·8 (11·9-30·8) | 24·8 (17·2-31·7) | 19·1 (10·7-29·8) |
| ADHD | 45 (6·6%) | 19 (4·8%) | 26 (9·0%) |
| Age at first record (year), cases | 22·7 (7·3-32·2) | 21·0 (7·1-29·4) | 23·0 (9·4-33·0) |
| Co-occurrence of ASD and ADHD | 53 (7·7%) | 21 (5·3%) | 32 (11·1%) |
| Age at first record (year), cases | 21·7 (6·5-32·1) | 21·0 (6·1-29·1) | 22·2 (7·1-32·7) |
| Any neuropsychiatric diagnosis | 150 (21·8%) | 80 (20·1%) | 70 (24·3%) |
| Age at first record (year), cases | 20·2 (7·0-32·0) | 21·9 (7·7-31·9) | 18·3 (6·6-31·5) |

^a^ Year of delivery; ^b^ Diagnosis recorded before and after delivery; ^c^ During pregnancy; ^d^ Gestational week 30;

Any maternal neuropsychiatric diagnosis defined as any mother with at least one primary diagnosis of any ICD10: F00-99 code; HbAA: Haemoglobin adducts of acrylamide; HbGA: Haemoglobin adducts of glycidamide; HbEO: Haemoglobin adducts of ethylene oxide; ADHD: attention-deficit/hyperactivity disorder; ASD: autism spectrum disorder. All cases are based on ICD8 and ICD10 diagnosis; cases of depression, anxiety, attention-deficit/hyperactivity disorder (ADHD), and co-existing autism spectrum disorder (ASD) and ADHD additionally include prescribed medication codes. HbGA has 56·3% missing observations.

**Table S4. Associations between prenatal exposure to acrylamide modelled as quartiles and the risk of developing neuropsychiatric disorders in the first 34 years of life (N=861).**

| Neuropsychiatric disorders (cases) |  | Acrylamide quartiles (Ref:Q1)  IRR (95% CI) | p-for trend |
| --- | --- | --- | --- |
| Depression (N=146) | Q2 | 1·06 (0·68, 1·66) | 0·077 |
|  | Q3 | 1·12 (0·70, 1·80) |  |
|  | Q4 | 1·78 (1·02, 3·11) |  |
|  |  |  |  |
| Anxiety (N=72) | Q2 | 0·67 (0·35, 1·29) | 0·80 |
|  | Q3 | 0·56 (0·27, 1·17) |  |
|  | Q4 | 1·35 (0·62, 2·97) |  |
| Any neuropsychiatric disorder (N=150) | Q2 | 1·00 (0·63, 1·60) | 0·138 |
|  | Q3 | 1·19 (0·74, 1·90) |  |
|  | Q4 | 1·57 (0·88, 2·78) |  |

Prenatal exposure to acrylamide modelled quartiles, with Q1 set as reference; models adjustment includes maternal active smoking, income, education, parity, sex, offspring’s gestational age, HbEO levels, alcohol intake and healthy eating index. All cases are based on ICD8 and ICD10 diagnosis; cases of depression and anxiety additionally include prescribed medication codes.

**Table S5. Associations between prenatal exposure to acrylamide and the risk of developing neuropsychiatric disorders in the first 34 years of life (N=861), offspring of nonsmokers (n=573) and offspring of women classified as active smokers during pregnancy (n=288).**

| Neuropsychiatric disorder | Population | | Crude Model  IRR (95 % CI) | | Basic Model  IRR (95 % CI) | | Further adjusted Model  IRR (95 % CI) |
| --- | --- | --- | --- | --- | --- | --- | --- |
| Depression | All | 1·36 (1·00, 1·87) | | 1·50 (1·00, 2·26) | | 1·62 (1·03, 2·54) | |
|  | Nonsmokers | 2·01 (1·04, 3·87) | | 1·88 (0·97, 3·66) | | 1·79 (0·89, 3·58) | |
|  | Smokers | 1·30 (0·81, 2·09) | | 1·35 (0·84, 2·18) | | 1·47 (0·82, 2·63) | |
|  |  |  | |  | |  | |
| Anxiety | All | 1·97 (1·29, 3·03) | | 2·03 (1·15, 3·58) | | 2·28 (1·22, 4·26) | |
|  | Nonsmokers | 1·80 (0·64, 5·04) | | 1·98 (0·69, 5·65) | | 2·11 (0·72, 6·23) | |
|  | Smokers | 2·09 (1·06, 4·13) | | 2·14 (1·08, 4·25) | | 2·22 (0·98, 5·00) | |
|  |  |  | |  | |  | |
| ADHD | All | 1·54 (0·87, 2·73) | | 1·12 (0·56, 2·23) | | 1·57 (0·73, 3·35) | |
|  | Nonsmokers | 1·77 (0·41, 7·60) | | 1·88 (0·43, 8·29) | | 1·90 (0·40, 9·14) | |
|  | Smokers | 1·05 (0·50, 2·20) | | 1·07 (0·51, 2·25) | | 1·67 (0·67, 4·17) | |
|  |  |  | |  | |  | |
| Co-occurrence of ASD and ADHD | All | 1·45 (0·85, 2·48) | | 0·97 (0·52, 1·83) | | 1·47 (0·72, 3·00) | |
|  | Nonsmokers | 1·60 (0·37, 6·81) | | 1·78 (0·41, 7·77) | | 1·62 (0·33, 7·99) | |
|  | Smokers | 0·90 (0·46, 1·75) | | 0·92 (0·47, 1·81) | | 1·58 (0·69, 3·65) | |
|  |  |  | |  | |  | |
| Any neuropsychiatric diagnosis | All | 1·45 (1·06, 1·99) | | 1·23 (0·82, 1·84) | | 1·40 (0·89, 2·21) | |
|  | Nonsmokers | 1·47 (0·68, 3·18) | | 1·54 (0·70, 3·36) | | 1·59 (0·70, 3·59) | |
|  | Smokers | 1·16 (0·73, 1·83) | | 1·18 (0·74, 1·86) | | 1·47 (0·84, 2·58) | |

Incidence risk ratio (IRR) and 95% confidence intervals (CI) from Poisson models of prenatal exposure to acrylamide modelled as dichotomized variable (high: ≥ 75^th^ percentile vs. low < the 75^th^ percentile of HbAA (pmol/g Hb), representing 146 pmol/g Hb in all population, pmol/g Hb in nonsmokers, and 204 pmol/g Hb in smokers). Crude Model A is adjusted for HbAA, Basic Model is adjusted for maternal smoking, income, education; Further adjusted model is further adjusted for sex, offspring’s gestational age, parity, HbEO levels, alcohol intake and healthy eating index. All cases are based on ICD8 and ICD10 diagnosis; cases of depression, anxiety, attention-deficit/hyperactivity disorder (ADHD), and co-existing autism spectrum disorder (ASD) and ADHD additionally include prescribed medication codes.

**Table S6. Hazard ratios for the associations between prenatal exposure to acrylamide and the risk of developing neuropsychiatric disorders in the first 34 years of life (N=861), offspring of nonsmokers (n=573) and offspring of women classified as active smokers during pregnancy (n=288)**

| Neuropsychiatric disorder | Population | Crude Model  HR (95% CI) | Basic Model  HR (95% CI) | Further adjusted Model  HR (95% CI) |
| --- | --- | --- | --- | --- |
| Depression | All | 1·39 (1·02, 1·90) | 1·54 (1·02, 2·32) | 1·74 (1·10, 2·74) |
|  | Nonsmokers | 2·12 (1·10, 4·09) | 1·97 (1·02, 3·84) | 1·93 (0·96, 3·90) |
|  | Smokers | 1·31 (0·82, 2·11) | 1·40 (0·87, 2·27) | 1·58 (0·88, 2·85) |
|  |  |  |  |  |
| Anxiety | All | 2·00 (1·30, 3·06) | 2·07 (1·17, 3·65) | 2·40 (1·28, 4·49) |
|  | Nonsmokers | 1·83 (0·65, 5·12) | 2·04 (0·71, 5·82) | 2·25 (0·76, 6·70) |
|  | Smokers | 2·10 (1·06, 4·14) | 2·16 (1·09, 4·28) | 2·27 (1·01, 5·15) |
|  |  |  |  |  |
| ADHD | All | 1·53 (0·87, 2·72) | 1·11 (0·56, 2·21) | 1·57 (0·74, 3·36) |
|  | Nonsmokers | 1·79 (0·42, 7·68) | 1·92 (0·44, 8·44) | 1·93 (0·40, 9·26) |
|  | Smokers | 1·03 (0·49, 2·16) | 1·05 (0·50, 2·20) | 1·70 (0·68, 4·23) |
|  |  |  |  |  |
| Co-occurrence of ASD and ADHD | All | 1·45 (0·85, 2·48) | 0·96 (0·51, 1·81) | 1·47 (0·72, 3·00) |
|  | Nonsmokers | 1·61 (0·38, 6·87) | 1·81 (0·41, 7·90) | 1·64 (0·33, 8·08) |
|  | Smokers | 0·88 (0·45, 1·72) | 0·90 (0·46, 1·77) | 1·58 (0·69, 3·63) |
|  |  |  |  |  |
| Any neuropsychiatric diagnosis | All | 1·47 (1·07, 2·01) | 1·23 (0·82, 1·85) | 1·44 (0·91, 2·27) |
|  | Nonsmokers | 1·50 (0·69, 3·24) | 1·57 (0·72, 3·44) | 1·63 (0·72, 3·69) |
|  | Smokers | 1·15 (0·73, 1·82) | 1·18 (0·74, 1·87) | 1·52 (0·87, 2·66) |

Hazard ratio (HR) and 95% confidence intervals (CI) from Cox models of prenatal exposure to acrylamide modelled as dichotomized variable (high: ≥ 75^th^ percentile vs. low < the 75^th^ percentile of HbAA (pmol/g Hb), representing 146 pmol/g Hb in all population, 96 pmol/g Hb in nonsmokers, and 204 pmol/g Hb in smokers.

Crude Model A is adjusted for HbAA, Basic Model is adjusted for maternal smoking, income, education; Further adjusted model is further adjusted for sex, offspring’s gestational age, parity, HbEO levels, alcohol intake and healthy eating index. All cases are based on ICD8 and ICD10 diagnosis; cases of depression, anxiety, attention-deficit/hyperactivity disorder (ADHD), and co-existing autism spectrum disorder (ASD) and ADHD additionally include prescribed medication codes.

**Table S7. Associations between prenatal exposure to acrylamide and mental health in the first 34 years of life - sensitivity analyses.**

|  | **Depression** | **Anxiety** | **ADHD** | **Co-occurrence of ASD and ADHD** | **Any neuropsychiatric diagnosis** |
| --- | --- | --- | --- | --- | --- |
|  | IRR (95% CI) | IRR (95% CI) | IRR (95% CI) | IRR (95% CI) | IRR (95% CI) |
| **Model with linear term** |  |  |  |  |  |
| Original dataset n=687 | 1·02 (0·99, 1·05) | 1·03 (0·99, 1·07) | 1·02 (0·97, 1·07) | 1·02 (0·98, 1·07) | 1·01 (0·97, 1·04) |
| Imputed dataset n=861 | 1·02 (0·99, 1·05) | 1·03 (0·99, 1·07) | 1·02 (0·97, 1·07) | 1·02 (0·98, 1·07) | 1·01 (0·98, 1·04) |
| Imputed dataset S1 n=861 linear | 1·02 (0·99, 1·05) | 1·03 (0·99, 1·06) | 1·01 (0·97, 1·06) | 1·02 (0·97, 1·06) | 1·01 (0·98, 1·04) |
| Imputed dataset S2 n=861 linear | 1·02 (0·99, 1·05) | 1·03 (0·99, 1·06) | 1·01 (0·97, 1·06) | 1·02 (0·97, 1·06) | 1·01 (0·98, 1·04) |
| Restricted to subset with HbGA n=363 | 1·02 (0·98, 1·05) | 1·03 (0·99, 1·07) | 1·02 (0·97, 1·08) | 1·02 (0·98, 1·08) | 1·00 (0·97, 1·04) |
| High HbGA levels n=363 | 1·01 (0·99, 1·04) | 1·01 (0·98, 1·05) | 0·99 (0·94, 1·04) | 0·99 (0·94, 1·04) | 1·01 (0·98, 1·03) |
| Excluding preterm cases n=824 | 1·02 (0·99, 1·05) | 1·03 (0·99, 1·07) | 1·01 (0·96, 1·07) | 1·02 (0·97, 1·06) | 1·00 (0·98, 1·03) |
| Excluding SGA cases n=861 | 1·02 (0·99, 1·05) | 1·01 (0·97, 1·05) | 1·02 (0·97, 1·07) | 1·02 (0·98, 1·07) | 1·00 (0·97, 1·03) |
| Excluding offsprings with any maternal neuropsychiatric disorders n=426 | 1·02 (0·97, 1·08) | 1·02 (0·95, 1·11) | 1·09 (1·01, 1·19) | 1·10 (1·01, 1·19) | 1·03 (0·97, 1·08) |
| Excluding samples from 37 gestation week n=858 | 1·02 (0·99, 1·05) | 1·02 (0·99, 1·06) | 1·01 (0·97, 1·06) | 1·01 (0·97, 1·06) | 1·01 (0·98, 1·03) |
| **Model with dichotomised term** (high ≥ 75^th^ vs low <75^th^) |  |  |  |  |  |
| Original dataset n=687 (≥P75) | 1·62 (1·00, 2·57) | 2·10 (1·08, 3·98) | 1·67 (0·76, 3·55) | 1·53 (0·73, 3·07) | 1·42 (0·89, 2·25) |
| Imputed dataset n=861 (≥P75) | 1·64 (1·05, 2·56) | 2·29 (1·23, 4·25) | 1·56 (0·73, 3·32) | 1·49 (0·73, 3·02) | 1·41 (0·90, 2·22) |
| Imputed dataset S1 n=861 (≥P75) | 1·56 (1·03, 2·37) | 1·89 (1·05, 3·42) | 1·67 (0·83, 3·35) | 1·56 (0·81, 3·00) | 1·34 (0·87, 2·07) |
| Imputed dataset S2 n=861 (≥P75) | 1·55 (1·02, 2·36) | 1·87 (1·04, 3·39) | 1·69 (0·85, 3·39) | 1·56 (0·81, 2·99) | 1·36 (0·88, 2·09) |
| Restricted to subset with HbGA n=363 (≥P75) | 1·59 (0·87, 2·91) | 2·67 (1·15, 6·22) | 1·93 (0·73, 5·13) | 1·71 (0·70, 4·15) | 1·82 (1·00, 3·32) |
| High HbGA levels n=363 (≥P75) | 1·78 (0·99, 3·21) | 1·40 (0·65, 3·02) | 1·03 (0·35, 3·02) | 0·76 (0·27, 2·09) | 1·24 (0·68, 2·25) |
| Excluding preterm cases n=824 (≥P75) | 1·57 (1·01, 2·44) | 2·09 (1·14, 3·85) | 1·46 (0·68, 3·12) | 1·38 (0·68, 2·81) | 1·32 (0·85, 2·06) |
| Excluding SGA cases n=861 (≥P75) | 1·53 (0·98, 2·39) | 1·38 (0·73, 2·64) | 1·93 (0·91, 4·11) | 1·74 (0·86, 3·53) | 1·26 (0·79, 2·01) |
| Excluding offsprings with any maternal neuropsychiatric disorders n=426 (≥P75) | 1·68 (0·80, 3·52) | 1·56 (0·53, 4·55) | 3·08 (0·96, 9·91) | 3·21 (1·08, 9·50) | 1·33 (0·61, 2·88) |
| Excluding samples from 37 gestation week n=858 (≥P75) | 1·54 (1·01, 2·35) | 1·83 (1·00, 3·33) | 1·63 (0·81, 3·27) | 1·53 (0·79, 2·95) | 1·31 (0·85, 2·02) |

Incidence risk ratio (IRR) and 95% confidence intervals (CI) from Poisson models of prenatal exposure to acrylamide modelled as dichotomized variable (high: ≥ 75^th^ percentile vs. low < 75^th^ percentile of HbAA (pmol/g Hb), representing 146 pmol/g Hb in all population).

Imputed dataset refers to the original dataset where missing data from covariates (n=174) was addressed using multiple imputation by chained equations.

Models are adjusted for maternal active smoking, income, education, parity, sex, offspring’s gestational age, HbEO levels, alcohol intake and healthy eating index; ADHD: attention-deficit/hyperactivity disorder; ASD: autism spectrum disorder. All cases are based on ICD8 and ICD10 diagnosis; cases of depression, anxiety, ADHD, and co-existing ASD and ADHD additionally include prescribed medication codes.

**Figure S1. DAG of confounders in prenatal acrylamide and neuropsychiatric disorders.**

**Figure S2. Flow-chart of the number of participants and reasons for attrition**

**Figure S3. Distribution of HbAA (pmol/g Hb; left; N=861) and HbGA (pmol/g Hb; right; N=363).**

**Figure S4. Upset plot of the relationship among neuropsychiatric disorders.**

Vertical bars represent the total intersection size; horizontal bars represent the total size of every disorder category and vertical dots represent the intersections among disorders

**Table S8. Associations between prenatal exposure to glycidamide and the risk of developing neuropsychiatric disorders in the first 34 years of life (N=363).**

| **Neuropsychiatric disorders** | **Crude Model**  **IRR (95% CI)** | **Basic Model**  **IRR (95% CI)** | **Further adjusted Model**  **IRR (95% CI)** |
| --- | --- | --- | --- |
| Depression | 1·49 (0·95, 2·33) | 1·67 (0·96, 2·89) | 1·78 (0·99, 3·21) |
| Anxiety | 1·52 (0·83, 2·78) | 1·39 (0·68, 2·81) | 1·40 (0·65, 3·02) |
| ADHD | 0·77 (0·31, 1·89) | 0·66 (0·25, 1·73) | 1·03 (0·35, 3·02) |
| Co-occurrence of ASD and ADHD | 0·62 (0·26, 1·50) | 0·51 (0·20, 1·30) | 0·76 (0·27, 2·09) |
| Any neuropsychiatric diagnosis | 1·31 (0·82, 2·08) | 1·08 (0·62, 1·86) | 1·24 (0·68, 2·25) |

Incidence risk ratio (IRR) and 95% confidence intervals (CI) from Poisson models of prenatal exposure to glycidamide modelled as dichotomized variable (high: ≥ 75^th^ percentile vs. low < the 75^th^ percentile of HbGA (pmol/g Hb), representing 230 pmol/g Hb

Crude Model A is adjusted for HbGA, Basic Model is adjusted for maternal smoking, income, education; Further adjusted model is further adjusted for sex, offspring’s gestational age, parity, HbEO levels, alcohol intake and healthy eating index. All cases are based on ICD8 and ICD10 diagnosis; cases of depression, anxiety, attention-deficit/hyperactivity disorder (ADHD), and co-existing autism spectrum disorder (ASD) and ADHD additionally include prescribed medication codes.

**Figure S5. Exposure-response curves of the associations between prenatal exposure to glycidamide and the risk of developing neuropsychiatric disorders.**

Incidence risk ratio (IRR) and 95% confidence intervals (CI) from Poisson models of prenatal exposure to glycidamide modelled continuously as natural spline with three degrees of freedom. Models were adjusted for maternal smoking, income, education, parity, sex, offspring’s gestational age, HbEO levels, alcohol intake and healthy eating index. ADHD: attention-deficit/hyperactivity disorder; ASD: autism spectrum disorder. All cases are based on ICD8 and ICD10 diagnosis; cases of depression, anxiety, attention-deficit/hyperactivity disorder (ADHD), and co-existing autism spectrum disorder and ADHD additionally include prescribed medication codes

**Table S9. Effect modification on the association between prenatal exposure to acrylamide and the risk of developing neuropsychiatric disorders in the first 34 years of life (N=861).**

|  | **Depression** | **Anxiety** | **ADHD** | **Co-occurrence of ASD and ADHD** | **Any neuropsychiatric diagnosis** |
| --- | --- | --- | --- | --- | --- |
| **Model** | IRR (95% CI) | IRR (95% CI) | IRR (95% CI) | IRR (95% CI) | IRR (95% CI) |
|  |  |  |  |  |  |
| Smoking, No | 1·98 (1·01, 3·88) | 2·03 (0·71, 5·79) | 1·42 (0·32, 6·26) | 1·36 (0·31, 5·92) | 1·55 (0·70, 3·40) |
| Smoking, Yes | 1·46 (0·84, 2·52) | 2·45 (1·12, 5·34) | 1·62 (0·67, 3·93) | 1·53 (0·68, 3·45) | 1·36 (0·80, 2·32) |
| Maternal Education, low | 2·15 (0·69, 6·67) | 0·68 (0·16, 2·86) | 1·58 (0·32, 7·67) | 1·73 (0·36, 8·30) | 0·69 (0·24, 2·00) |
| Maternal Education, middle | 1·72 (0·95, 3·11) | 3·03 (1·32, 6·96) | 0·88 (0·29, 2·74) | 0·74 (0·25, 2·20) | 1·39 (0·76, 2·55) |
| Maternal Education, high | 1·51 (0·85, 2·69) | 2·37 (1·08, 5·22) | 2·37 (0·90, 6·26) | 2·45 (1·02, 5·90) | 1·72 (0·96, 3·08) |
| Maternal neuropsychiatric disorders, No | 1·55 (0·97, 2·48) | 2·03 (1·04, 3·99) | 1·56 (0·70, 3·46) | 1·43 (0·67, 3·05) | 1·41 (0·87, 2·29) |
| Maternal neuropsychiatric disorders, Yes | 2·19 (0·99, 4·86) | 2·19 (0·77, 6·27) | 1·53 (0·41, 5·68) | 1·54 (0·46, 5·14) | 1·43 (0·64, 3·22) |
| Sex, Female | 1·66 (1·00, 2·74) | 2·30 (1·15, 4·61) | 1·10 (0·38, 3·23) | 1·28 (0·46, 3·52) | 1·55 (0·89, 2·70) |
| Sex, Male | 1·62 (0·84, 3·12) | 2·23 (0·89, 5·58) | 1·93 (0·79, 4·73) | 1·68 (0·73, 3·86) | 1·26 (0·70, 2·29) |

Incidence risk ratio (IRR) and 95% confidence intervals (CI) from Poisson models of prenatal exposure to acrylamide modelled as dichotomized variable (high: ≥ 75^th^ percentile vs. low < the 75^th^ percentile of HbAA (pmol/g Hb), representing 146 pmol/g Hb.

Models were adjusted for maternal smoking, income, education, parity, sex, offspring’s gestational age, HbEO levels, alcohol intake and healthy eating index. All cases are based on ICD8 and ICD10 diagnosis; cases of depression, anxiety, attention-deficit/hyperactivity disorder (ADHD), and co-existing autism spectrum disorder (ASD) and ADHD additionally include prescribed medication codes; *p-value for interaction (p<0·05)

**References:**

Bliddal M, Broe A, Pottegård A, Olsen J, Langhoff-Roos J. (2018). The Danish Medical Birth Register. Eur J Epidemiol. 33(1):27-36.

Bjerregaard, A. A., Halldorsson, T. I., Tetens, I., & Olsen, S. F. (2019). Mother’s dietary quality during pregnancy and offspring’s dietary quality in adolescence: Follow-up from a national birth cohort study of 19,582 mother-offspring pairs. PLoS Medicine, 16(9), e1002911. https://doi.org/10.1371/journal.pmed.1002911

Chandia-Poblete, D., Tuffier, S., Vryonidis, E., Halldorsson, T. I., Bjerregaard, A. A., Rytter, D., Bech, B. H., Henriksen, T. B., Olsen, S. F., Törnqvist, M., & others. (2025). Prenatal Exposure to Acrylamide, Fetal Growth and Newborn Size: A Biomarker-based Cohort Study from Denmark. Environmental Research, 122996.

Knudsen LB, Olsen J. The Danish Medical Birth Registry. Dan Med Bull. 1998 Jun;45(3):320-3. PMID: 9675544.

Lynge E, Sandegaard JL, Rebolj M. The Danish National Patient Register. Scand J Public Health. 2011 Jul;39(7 Suppl):30-3. doi: 10.1177/1403494811401482. PMID: 21775347.

Kildemoes HW, Sørensen HT, Hallas J. The Danish National Prescription Registry. Scand J Public Health. 2011 Jul;39(7 Suppl):38-41. doi: 10.1177/1403494810394717. PMID: 21775349.

Mors O, Perto GP, Mortensen PB. (2011). The Danish Psychiatric Central Research Register. Scand J Public Health. 39(7 Suppl):54-7.

Olsen, S., Hansen, H. S., Secher, N. J., Jensen, B., & Sandström, B. (1995). Gestation length and birth weight in relation to intake of marine n-3 fatty acids. British Journal of Nutrition, 73(3), 397–404.

Schmidt M, Schmidt SA, Sandegaard JL, Ehrenstein V, Pedersen L, Sørensen HT. (2015). The Danish National Patient Registry: a review of content, data quality, and research potential. Clin Epidemiol. 7:449-90.

Pedersen, M., von Stedingk, H., Botsivali, M., Agramunt, S., Alexander, J., Brunborg, G., Chatzi, L., Fleming, S., Fthenou, E., Granum, B., Gutzkow, K. B., Hardie, L. J., Knudsen, L. E., Kyrtopoulos, S. A., Mendez, M. A., Merlo, D. F., Nielsen, J. K., Rydberg, P., Segerbäck, D., … Kogevinas, M. (2012). Birth weight, head circumference, and prenatal exposure to acrylamide from maternal diet: The European prospective mother-child study (NewGeneris). Environmental Health Perspectives, 120(12), 1739–1745. https://doi.org/10.1289/ehp.1205327

Pottegård A, Schmidt SAJ, Wallach-Kildemoes H, Sørensen HT, Hallas J, Schmidt M. (2017). Data Resource Profile: The Danish National Prescription Registry. Int J Epidemiol. 46(3):798-798f.
